# The Impact of Aging on the Tongue Proteome

**DOI:** 10.3390/muscles5040073

**Published:** 2026-10-09

**Authors:** Kay Ohlendieck, Margit Zweyer, Felix Nebeling, Dieter Swandulla, Paul Dowling

**Affiliations:** 1 Department of Biology, Maynooth University, Co. Kildare, W23 V5XH Maynooth, Ireland; kay.ohlendieck@mu.ie; 2 Kathleen Lonsdale Institute for Human Health Research, Maynooth University, Co. Kildare, W23 V5XH Maynooth, Ireland; 3 German Centre for Neurodegenerative Diseases, 53103 Bonn, Germany; margit.zweyer@dzne.de; 4 Department of Neurooncology, Center for Neurology, University Hospital Bonn, 53127 Bonn, Germany; felix.nebeling@dzne.de; 5 Institute of Physiology, Faculty of Medicine, University of Bonn, 53151 Bonn, Germany; swandulla@uni-bonn.de

**Keywords:** biomarker, mass spectrometry, muscle proteomics, sarcopenia of old age, tongue

## Abstract

The tongue is a highly important, multifunctional, and complex organ in human physiology. It is critical for digestion, speech, immune surveillance, and sensory perception, playing a dynamic role in daily life. Abnormalities in the innervation and/or myofiber integrity of the tongue can have severe pathophysiological effects on swallowing, speaking, chewing and bodily balance. The age-related decrease in skeletal muscle mass and contractile strength, a recognized risk factor for sarcopenia and frailty, also impacts the tongue muscles. This report has examined the tongue proteome and evaluated changes in protein abundance associated with the aging phenotype. Interestingly, the proteomic cataloging of the contractile apparatus of tongue muscle identified myosin heavy chain isoform MyHC-13, which is the superfast type of myosin that is restricted to specialized skeletal muscles such as extraocular and laryngeal muscles. Proteomics revealed that tongue aging is associated with considerable changes in the protein expression pattern. Decreases were identified in membrane repair-associated annexin A isoforms, luminal calcium-binding proteins, contractile proteins, and cytoskeletal elements. Lower levels of fast myosin isoform MyHC-2B suggests potential age-associated fiber type shifting towards slower twitch properties. Elevated levels of the fibrosis marker collagen COL-VI, which increased deposition in aged tongue muscle was verified by immunofluorescence microscopy, agrees with the idea of a higher degree of susceptibility to reactive myofibrosis in sarcopenia of old age.

## 1. Introduction

The tongue is a multifunctional organ in the oral cavity that is anatomically divided into the root structure (*radix lingue*) and the main body (*corpus lingue*) consisting of paired muscles and a mucosal layer with various types of papillae [1,2,3]. Crucial functions of the tongue are linked to body-wide processes, including digestion, autocrine/paracrine mechanisms, and speech. As an accessory organ of the sensory and digestive systems, the tongue is intrinsically involved in taste sensations, mastication, and swallowing, as well as clearing of the oral cavity [4]. Importantly, as a barrier organ of the oral cavity and being part of the protective oral mucosa, the tongue functions in immune surveillance [5] to defend the body from potentially harmful microbial, environmental and/or food-associated antigenic substances via both innate and adaptive immune responses [6,7,8]. Of note, tongue posture and pressure against the palate may activate trigeminal nerve sensory pathways, which are involved in postural control and balance [9].

The tongue can be associated with both superficial lesions and a variety of distinct disorders [10], and is also critical for clinical diagnosis due to the fact that the examination of the tongue can reveal specific lingual alterations [11] that may mirror disease status [12]. Tongue diseases include lingual pain, abnormal lingual sensitivity and impaired taste sensation [13], as well as tongue squamous cell carcinoma [14] and a variety of movement disorders such as overactive involuntary muscle twitching (fasciculations), uncontrolled choreic tongue movements (harlequin tongue), orolingual tremor and dystonia of the tongue [15]. During natural aging, tongue muscle physiology was shown to be impaired. Measurement of tongue pressure showed a significant decline in tongue muscle strength in sarcopenic individuals [16,17] and the fat mass of the tongue was shown to significantly increase with aging [18]. Sarcopenia of old age is defined as the progressive loss of skeletal muscle mass and contractile strength in the elderly [19,20,21] and is associated with changes in both myofiber size and fiber type distribution [22,23,24,25]. In analogy to general changes observed in the aged neuromuscular system, tongue muscle aging was shown to be associated with loss of tissue integrity, decreased contractile strength resulting in dysphagia, stem cell exhaustion, bioenergetic alterations, fiber type shifting, myofibrosis and fragmentation of the neuromuscular junction [26,27,28,29,30,31,32,33,34].

Although lingual muscles are crucial for eating and speech, relatively few studies have focused on the effects of aging on tongue musculature, as reviewed by Doyle et al. [5]. It was therefore of interest to study potential proteome-wide changes in the aged tongue using comparative mass spectrometry (MS) to survey young versus aged tongue. In contrast to the proteomic cataloging of a large number of individual skeletal muscles from animals and humans [35,36,37,38,39], including the screening of aged voluntary striated muscles [40,41,42,43,44], only limited numbers of proteomic studies have focused on the tongue, including the optimization of tissue lysis prior to proteomic analysis [45], the comparative analysis of tongue cancer and non-cancerous mucosa [46,47,48], and the effects of a Western diet on the remodeling of the tongue proteome [49].

Thus, in analogy to the recently outlined efforts for the systematic determination of cellular diversity involved in feeding, communication and breathing in form of the Human Oral and Craniofacial Cell Atlas [50], which also aims at the single cell transcriptomic characterization of tongue tissue [51], this report focuses on the protein cataloging of the tongue proteome and its aged phenotype. Here, we have employed bottom-up proteomics to characterize the mouse tongue and establish its protein constellation since rodent model systems are widely used in neuromuscular research [52,53,54] including investigations into the pathobiochemical mechanisms that underlie tissue wasting diseases and sarcopenia of lingual muscles [55,56,57].

## 2. Results

The primary finding of this report is that MS-based proteomics can be used to both efficiently catalog tongue muscle specimens and determine protein expression changes within a pathophysiological context. In contrast to screening methods such as semi-quantitative immunoblotting, which is heavily limited by the availability and specificity of antibodies and therefore of limited use for the identification of dynamic proteoforms [58], large-scale proteomic surveys represent a more cost-effective and less labor-intensive technique. This is especially relevant for studying the skeletal muscle proteome, which is characterized by a relatively skewed abundance distribution of abundant contractile protein species and also exhibits a high dynamic range of proteoform expression and post-translational modifications [35,36,37,38,39].

Proteomic profiling of the tongue has identified the expression of a variety of muscle- proteins that are associated with the sarcomere, specifically components of the contractile apparatus, the auxiliary titin filament, the Z-disc and the M-line, and markers of distinct subcellular locations, including the extracellular space, the extracellular matrix (epimysium, endomysium, perimysium, basal lamina), the plasmalemmal adhesion complex and costameres, the sarcolemma with its caveolae and sarcolemmal repair system, transverse tubules, the membrane cytoskeleton, intermediate filaments, microtubules, the sarcoplasmic reticulum (longitudinal tubules, terminal cisternae, lumen), triad junction membrane, ribosomes, Golgi apparatus, lysosome, peroxisome, proteasome, myonuclei, mitochondria and the cytosol [59,60,61].

Specific proteoforms include markers of the actomyosin apparatus, the regulators of excitation-contraction coupling, molecular chaperones, bioenergetic enzymes and organelle-specific proteins. The proteomic map of the mouse tongue muscles can now be employed as a reference for future large-scale investigations of systemic physiological versus pathological changes in the tongue. To illustrate the bioanalytical potential of proteomic screening, the aged phenotype of tongue muscle has been studied, and the mass spectrometric findings are discussed in this report.

### 2.1. Bottom-Up Proteomic Profiling of Tongue Muscle

#### 2.1.1. Bioinformatic Analysis of Protein Class Distribution in the Tongue Proteome

**Figure 1:**
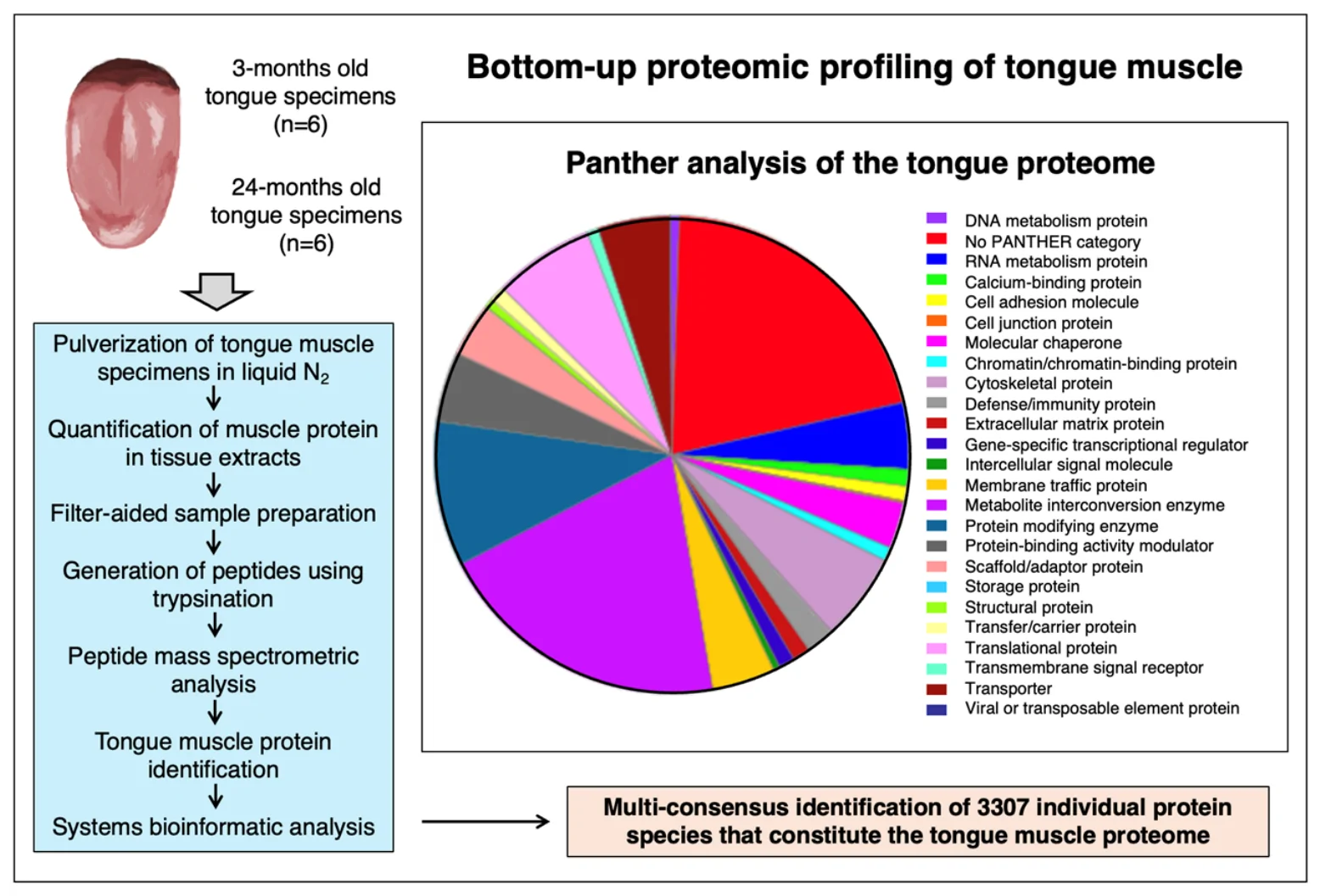
Overview of the bioanalytical strategy to determine the tongue proteome. The experimental workflow and the findings from the bioinformatic PANTHER (http://pantherdb.org) [62] analysis of protein classes in the tongue proteome are shown.

Following the survey of crude tissue extracts from mouse tongue using a Thermo Fisher Ascend mass spectrometer coupled with a NeoVanquish (Thermo Fisher Scientific, Hemel Hempstead, UK) liquid chromatography (LC) system for label-free LC-MS/MS, the list of over 3000 identified protein species was further analyzed by bioinformatic PANTHER (release 19.0) [62] analysis to determine the protein class distribution of the tongue proteome. Figure 1 gives an overview of the bioanalytical approach used to characterize the tongue and the results from the PANTHER analysis.

The main protein classes related to DNA metabolism, RNA metabolism, calcium-binding, cell adhesion, cell junction formation, molecular chaperoning, chromatin/chromatin-binding, the cytoskeleton, defense/immunity, the extracellular matrix, gene-specific transcriptional regulation, intercellular signaling, membrane trafficking, metabolite interconversion, protein modifications, protein-binding modulation, scaffolding/adaptations, storage, structural maintenance, transfer/carrier activity, translation, transmembrane signaling and transport.

#### 2.1.2. Proteomic Identification of Major Sarcomeric Tongue Muscle Proteins

The bottom-up proteomic profiling of total extracts from mouse muscle has identified a large number of contractile proteins and their regulatory components belonging to the A-band, I-band, titin filament, Z-disc and M-line region [63]. As listed in Table 1, key contractile proteins of the myosin-containing thick filament were identified in the form of a large number of myosin heavy chains (MyHC) and light chains (MLC) in both 3-month and 24-month old tissue preparations. The myosin heavy chain (MyHC) repertoire included Myosin-1 to Myosin-4, which represents the fast isoforms MyHC-2X (*Myh1* gene), MyHC-2A (*Myh2* gene) and MyHC-2B (*Myh4* gene), as well as the embryonic isoform MyHC-3 (*Myh3* gene). A differential identification of Myosin-6, the cardiac (α) MyHC-6 (*Myh6* gene) isoform, and Myosin-7, the cardiac (β) MyHC-1 (*Myh7* gene) isoform, was observed in young versus aged tongue muscle. Both preparations from young and aged tongue contained Myosin-8, the perinatal MyHC-8 (*Myh8* gene) isoform.

Interestingly, Myosin-13, the superfast type of myosin that is highly expressed in specialized skeletal muscles such as extraocular myofibers [64] and laryngeal muscles [65], was also identified in tongue muscle as the MyHC-13 isoform (*Myh13* gene). Major myosin light chains (MLC) were determined to be present in tongue muscle, including MLC1/3 (essential/fast MLC), MLC3 (essential/slow MLC) and MLC11 (regulatory/fast MLC). In addition, myosin light chain kinase MYLK2 and the myosin binding proteins MyBP-C (slow) and MyBP-C (fast) were identified by proteomics [63].

The major contractile protein of the thin filament in tongue muscle was identified as α-actin in conjunction with its regulatory complex consisting of the troponin subunits TnC, TnI and TnT and tropomyosin isoforms TPM1, TPM2, TPM3 and TPM4. Additional actin filament associated components included the F-actin-capping proteins CapZ-A1, CapZ-A2 and CapZ-B, as well as the tropomodulins TMOD1 and TMOD3, the Xin actin-binding protein XIRP and myopalladin.

The giant protein component of the thin filament, nebulin of apparent 871 kDa and its anchoring protein NRAP, in addition to the extremely high-molecular-mass protein titin of apparent 3925 kDa and its associated ankyrin repeat protein MARP/ANKRD2, were clearly identified by MS analysis [66]. The tongue muscle proteome was also shown to contain key sarcomeric proteins of the Z-disc, such as actinin-2, myozenin MYOZ1, muscle-type filamin-C and telethonin, and the M-line, including obscurin of apparent 967 kDa and the myomesin isoforms MYOM1, MYOM2 and MYOM3 (Table 1).

#### 2.1.3. Proteomic Identification of Subcellular Tongue Marker Proteins

Following the detailed analysis of the sarcomeric protein constellation, subcellular markers of tongue muscles were established. Table 2 lists proteomic marker proteins that were identified by MS analysis. The proteomic survey lists serum albumin and hemoglobin as components of the extracellular space and external oxygen supply of skeletal muscle tissue, respectively.

Key proteins of the main layers of the extensive extracellular matrix of tongue muscle, the epimysium, endomysium and perimysium, were classified as collagen COL-I, COL-VI and decorin, respectively, plus laminin of the basal lamina [67]. As markers of the sarcolemmal adhesion complex, costameres, the sarcolemma membrane, caveolae structures and the sarcolemmal repair system are listed integrin, talin, the Na^+^/K^+^-ATPase, caveolin CAV1 and dysferlin, respectively.

The various parts of the intracellular cytoskeleton are represented by spectrin of the membrane cytoskeleton, desmin of the intermediate filaments and tubulin of the microtubules [59,60,61,62]. Internal membrane systems, such as transverse tubules, the longitudinal tubules, terminal cisternae and lumen of the sarcoplasmic reticulum and the triad junctions are linked to the voltage-dependent L-type Ca^2+^-channel, sarcalumenin, the fast SERCA1 isoform of the sarcoplasmic reticulum Ca^2+^-ATPase, the fast CSQ1 isoform of the Ca^2+^-binding protein calsequestrin, and the RYR1 isoform of the ryanodine receptor Ca^2+^-release channel [59,60,61], respectively.

Common markers of organelles are listed as 40S ribosomal protein S4 (ribosomes), the general vesicular transport factor (Golgi apparatus), the lysosome-associated membrane glycoprotein LAMP1 (lysosomes), catalase (peroxisomes), the ubiquitin-conjugating enzyme E2 (proteasomes) and emerin (nuclei). Table 2 displays the mitochondrial proteins named voltage-dependent anion-selective channel protein VDAC1, the MIC60 subunit of the mitochondrial contact site complex, NADH dehydrogenase, succinate dehydrogenase, the cytochrome b-c complex, cytochrome c oxidase, ATP synthase, isocitrate dehydrogenase isoform IDH2 and hexokinase isoform HK2 as markers of the outer membrane, contact sites, inner membrane complex I, inner membrane complex II, inner membrane complex III, inner membrane complex IV, inner membrane complex V, the matrix and the metabolic flux regulator system between mitochondria and the cytosol [59,60,61]. Marker proteins of the sarcosol include phosphofructokinase of the glycolytic pathway, fatty acid binding protein isoform FABP3/FABPH involved in metabolite transportation, myoglobin responsible for intracellular oxygen transport and the cardiovascular heat shock protein isoform HSPB7 that plays a key role in the cellular stress response of skeletal muscle tissues (Table 2).

### 2.2. Comparative Proteomic Profiling of Young Versus Aged Tongue Muscle

#### 2.2.1. Bioinformatic PANTHER Analysis of Proteome-Wide Changes During Tongue Aging

**Figure 2:**
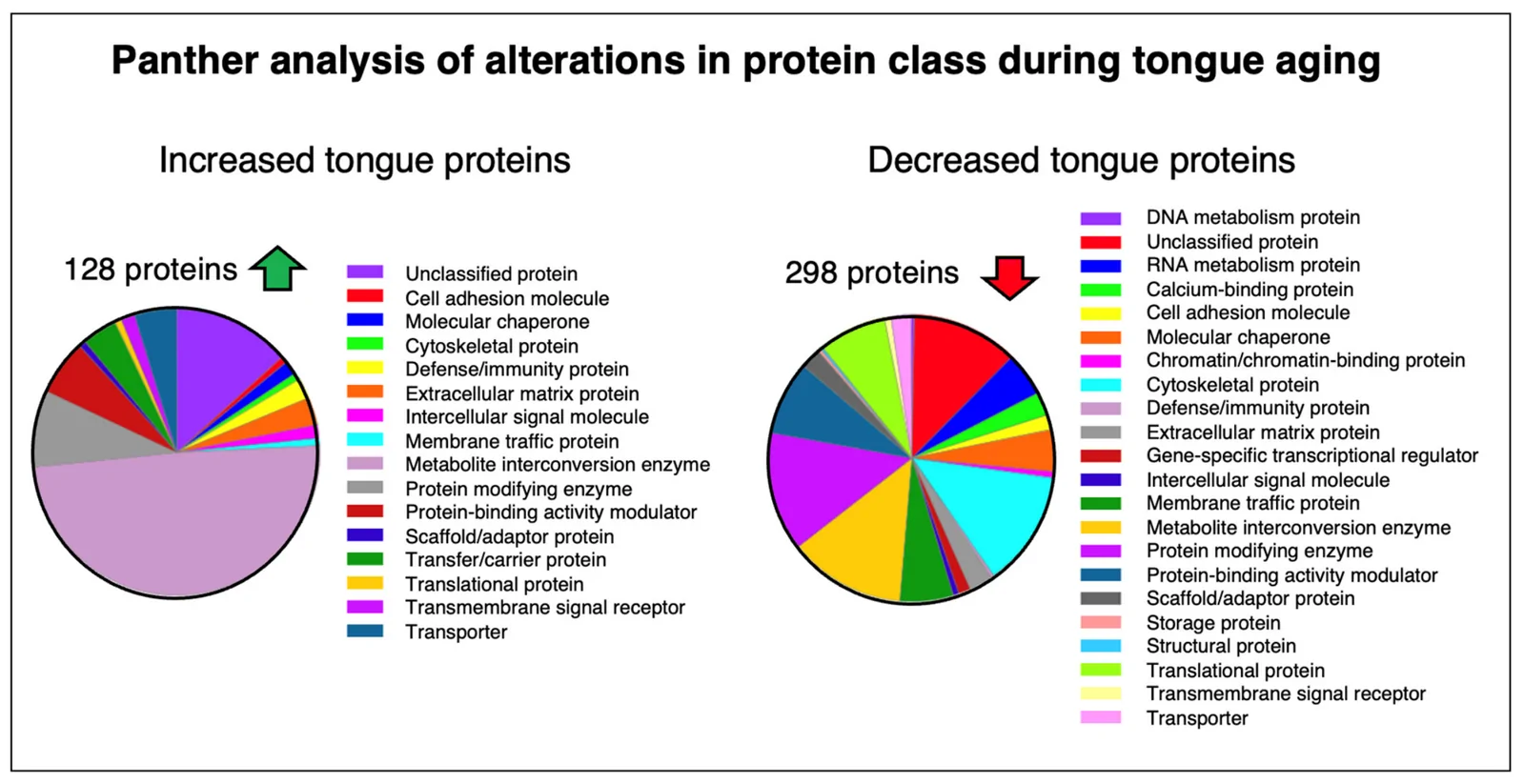
Comparative bioinformatic PANTHER (http://pantherdb.org) [62] analysis of protein classes with an increased versus decreased abundance in the aged mouse tongue. 128 proteins increased in abundance (green arrow) and 298 proteins decreased in abundance (red arrow) during tongue aging.

Proteomics identified changes in a large number of proteins during aging of the mouse tongue, i.e., 128 increased proteins and 298 decreased proteins (see Supplementary Data). Figure 2 shows the bioinformatic PANTHER [62] analysis of altered protein classes in senescent tongue tissue.

The main types of proteins with an increased abundance belong to the class of cell adhesion molecules, extracellular matrix proteins, metabolite interconversion enzymes, protein modifying enzymes, transfer/carrier proteins, and transporters. Increased proteins are involved in DNA metabolism, RNA metabolism, molecular chaperoning, membrane trafficking, metabolite interconversion, protein-binding activity modulation, structural maintenance, and translation.

#### 2.2.2. Heatmap and Listing of Proteome-Wide Changes During Tongue Aging

**Figure 3:**
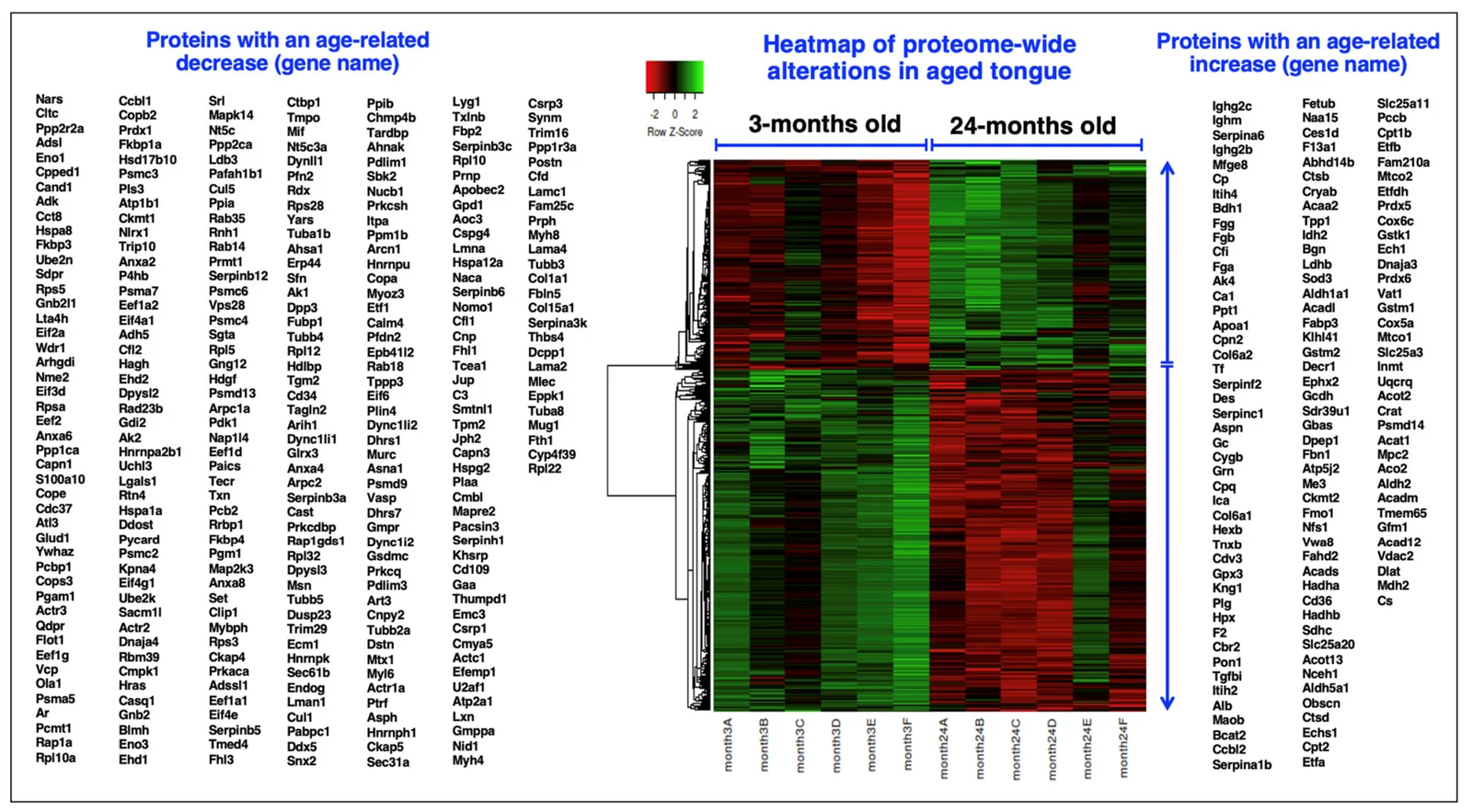
Heatmap and listing of proteome-wide changes during tongue aging.

A heatmap of changes in the protein constellation of the aged tongue is provided in Figure 3, which also includes a listing of decreased versus increased proteins symbolized by their respective gene names. Interesting proteomic changes include the reduction in annexin isoforms ANXA2, ANXA4 and ANXA6, the luminal calcium-binding proteins sarcalumenin and calsequestrin, various isoforms of tubulin, matrisomal proteins such as collagen isoforms COL-I and COL-XV, and the contractile apparatus components myosin binding protein MYBPH and fast MyHC-2B (Myosin-4). These alterations suggest age-associated modifications in membrane repair mechanisms, calcium handling, the provision of cytoskeletal networks, the maintenance of the extracellular matrix, and potential fiber type shifting towards slower twitch properties. On the other hand, increases in the fibrosis markers collagen COL-VI, fibrinogen and asporin suggest increased collagen deposition and a higher degree of susceptibility to reactive myofibrosis in in the aged tongue. The identification of the small heat shock protein αB-crystallin in the senescent tongue suggests cellular stress during aging. Overall, the heatmap visualizes that tongue aging is associated with considerable changes in the protein expression pattern of this highly specialized muscular organ.

#### 2.2.3. Bioinformatic ShinyGO Analysis of Alterations in Biological Processes During Tongue Aging

**Figure 4:**
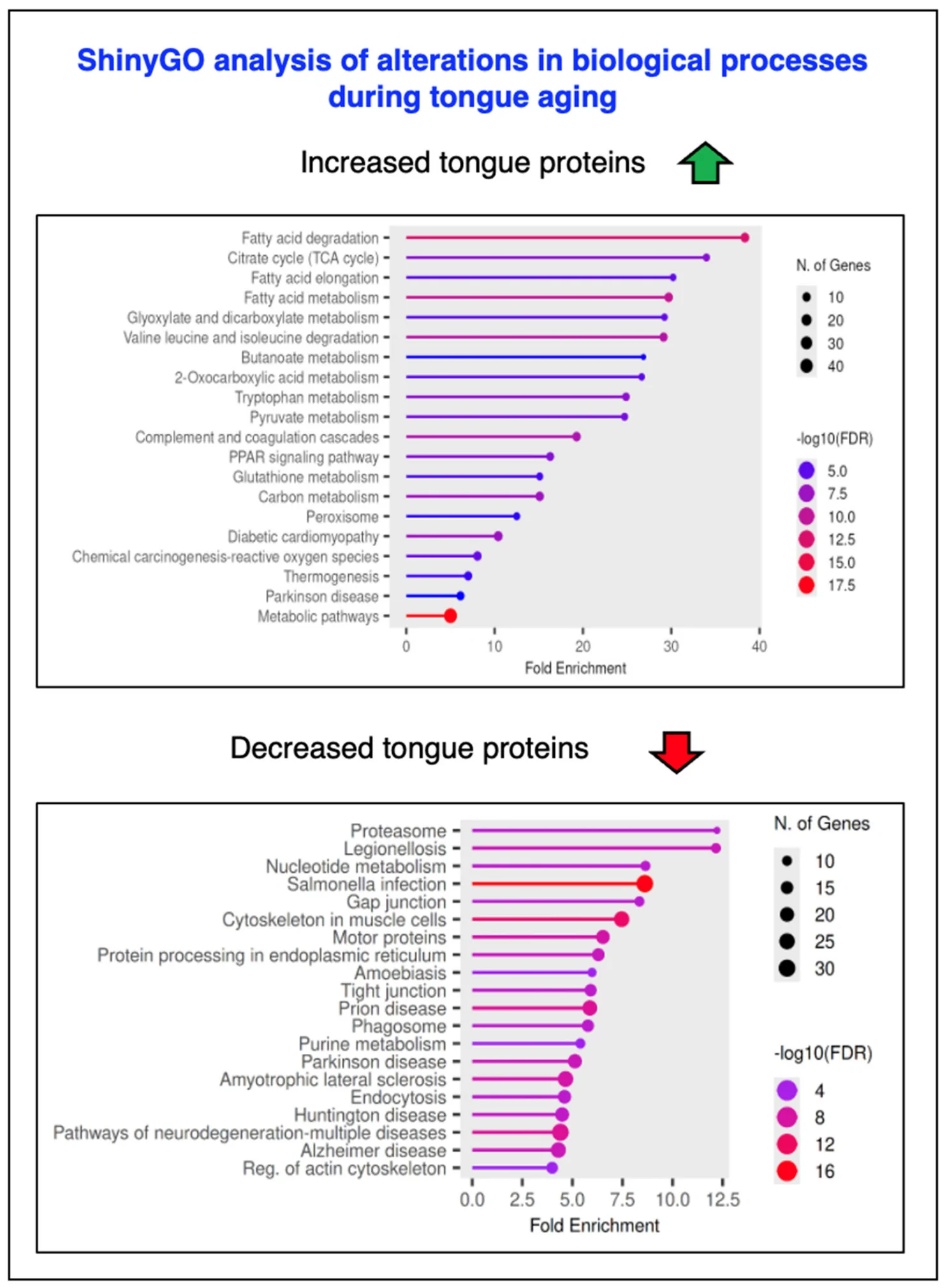
Bioinformatic ShinyGO (http://bioinformatics.sdstate.edu/go77/; accessed on 1 March 2026) [68] analysis of alterations in biological processes during tongue aging. The enrichment analysis was performed using an FDR cutoff of q = 0.05; the size of the circle represents the number of responsible genes, with larger circles representing more genes. The color represents the strength of the FDR, with red and blue indicating higher and lower FDR values, respectively.

The findings from the bioinformatic ShinyGO (version 0.75) [68] analysis of alterations in biological processes during tongue aging are shown in Figure 4. The KEGG (Kyoto Encyclopedia of Genes and Genomes) [69] pathway comparison indicates increases versus decreases in specific processes related to fatty acid metabolism, the citric acid cycle, amino acid degradation, pyruvate metabolism, glutathione metabolism, carbon metabolism, nucleotide metabolism and purine metabolism, as well as changes in proteasome, phagosomes, endocytosis, cytoskeletal maintenance, motor protein abundance and neurodegenerative pathways.

#### 2.2.4. Bioinformatic STRING Analysis of Potential Protein–Protein Interaction Changes During Tongue Aging

**Figure 5:**
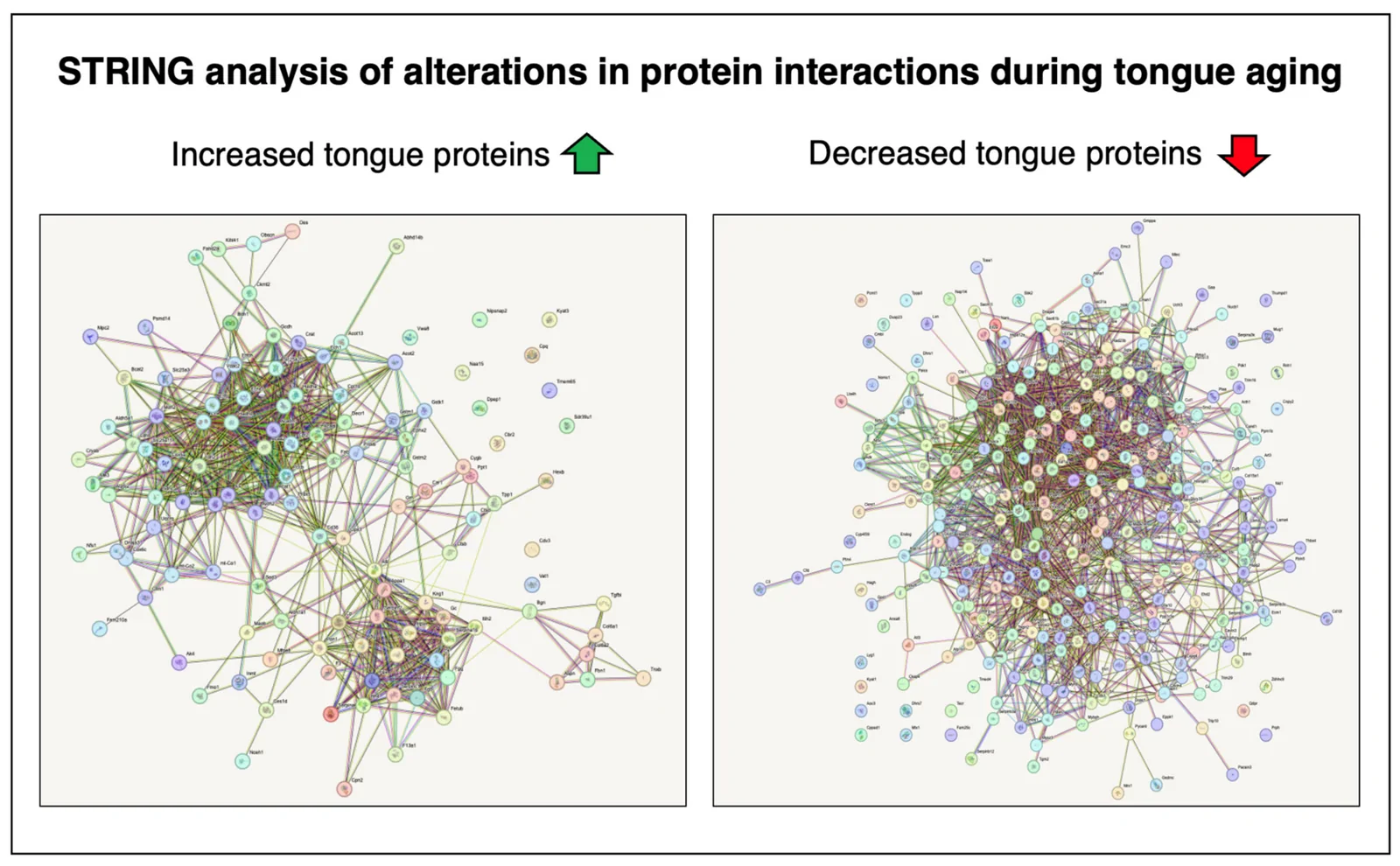
Bioinformatic STRING (https://string-db.org) [70] analysis of alterations in protein–protein interactions during tongue aging. The figure shows the STRING network divided into two clusters identified by k-means clustering for proteins increased in abundance in aging tongue muscle. These clusters were associated with metabolism (larger cluster) and signaling. Different colors of the lines represent the types of evidence for association: neighborhood (green), gene fusion (red), cooccurrence (blue), co-expression (dark), experiments (pink), databases (sky blue).

Identified tongue proteins with an altered abundance in young versus aged tissue specimens were analyzed by the bioinformatic STRING (version 12.5) [70] program to determine potential alterations in predicted protein–protein interaction patterns during muscle aging (Figure 5). The diagram below shows clusters of hubs of protein interaction networks that might be altered during tongue aging. The increases in the interaction hub of extracellular matrix proteins are interesting, especially the COL-VI isoform of collagen [67] that might indicate a higher susceptibility to myofibrosis in the aged tongue.

#### 2.2.5. Overview of Proteome-Wide Changes During Tongue Aging

The MS-based proteomic screening of young versus aged tongue preparations revealed a large number of increased versus decreased protein species. Table 3 and Table 4 list a select number of proteomic hits with a cutoff of a 1.5-fold change in abundance. Interesting proteome-wide changes include the increased expression of proteins involved in energy metabolism, hormonal regulation, iron homeostasis, inflammation, collagen accumulation, mitochondrial flexibility, nucleotide homeostasis, muscle regeneration, carbon dioxide removal, antioxidative protection, glucose metabolism and the maintenance of the network of intermediate filaments.

Interesting proteome-wide changes include the decreased expression of proteins involved in matrisomal modifications, cytoskeletal rearrangements, muscle regeneration, protein glycosylation, bioenergetic processes, antioxidant defense, inflammatory response, iron transport, detoxification, and the regulation of blood flow.

#### 2.2.6. Histological and Immunofluorescence Microscopical Analysis of Tongue Aging

**Figure 6:**
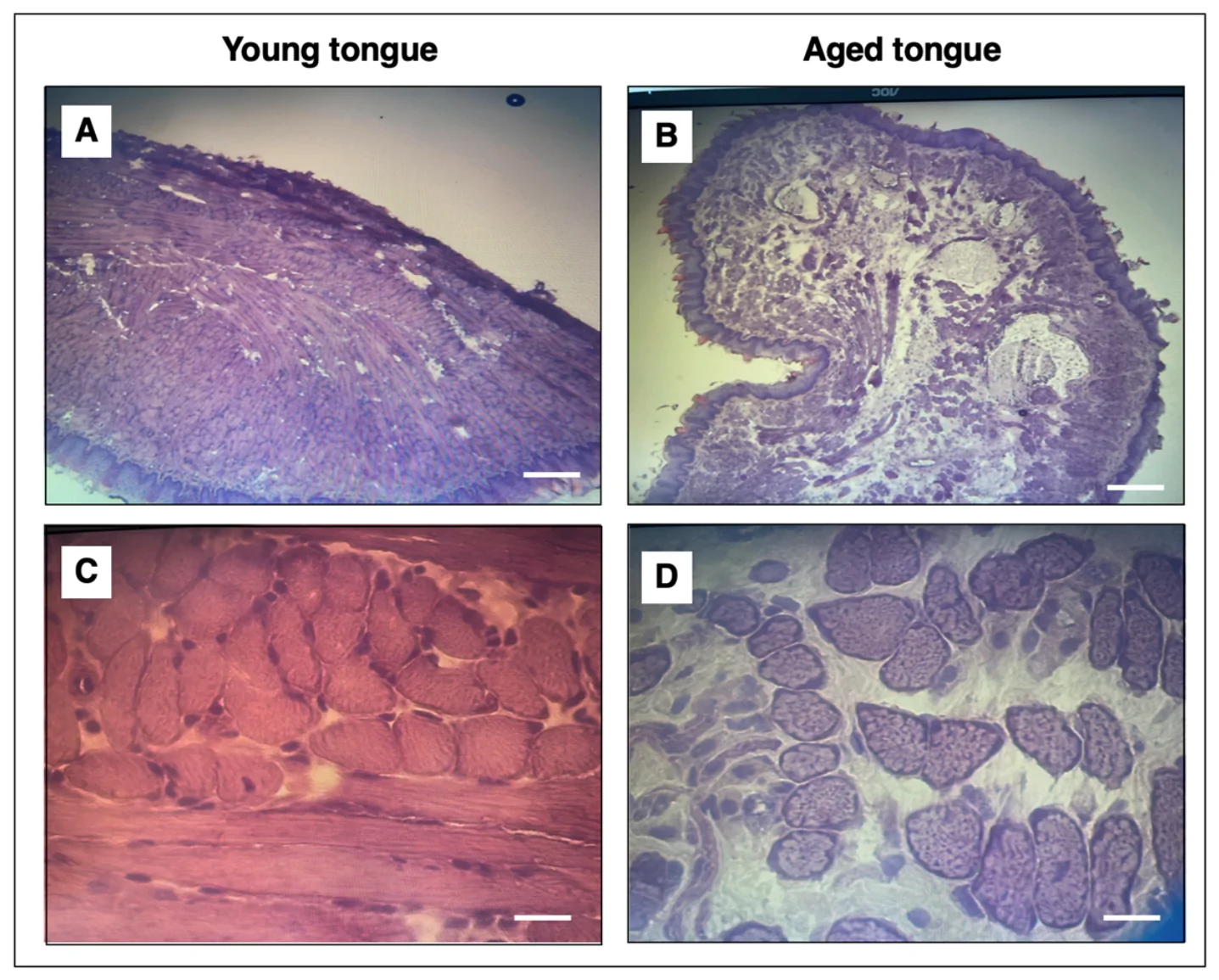
Histological comparison of young versus aged tongue. Bars equal 100 μm in (**A**,**B**) and 25 μm in (**C**,**D**). A sizable increase in myofiber degeneration and apparent replacement by fibrous structures and fatty tissue in aging tongue muscle is visible.

Following the proteomic and bioinformatic analysis of the effects of aging on the tongue, a histological and immunofluorescence microscopical analysis was conducted. In general, bioimaging and cell biological studies have established that skeletal muscle aging is associated with progressive myofiber degeneration, inflammation and decreased contractile strength, and a concomitant increase of non-contractile cell types, including intramuscular adipose and connective tissues [19]. The fibro-fatty replacement and infiltration of contractile tissues contributes to detrimental patterns of myosteatosis and myofibrosis, which in turn results in loss of intrinsic skeletal muscle strength in the elderly. The pathophysiological replacement of contractile fibers by non-functional fibrous and adipose tissue is often progressive, chronic, and irreversible [71,72,73]. The histological analysis of young versus aged tongue tissue confirmed this pattern of apparent fibro-fatty changes during aging in this specialized muscle system. Figure 6 illustrates a drastic increase in myofiber degeneration and apparent replacement by fibrous structures and fatty tissue in 24-month old mouse tongue.

**Figure 7:**
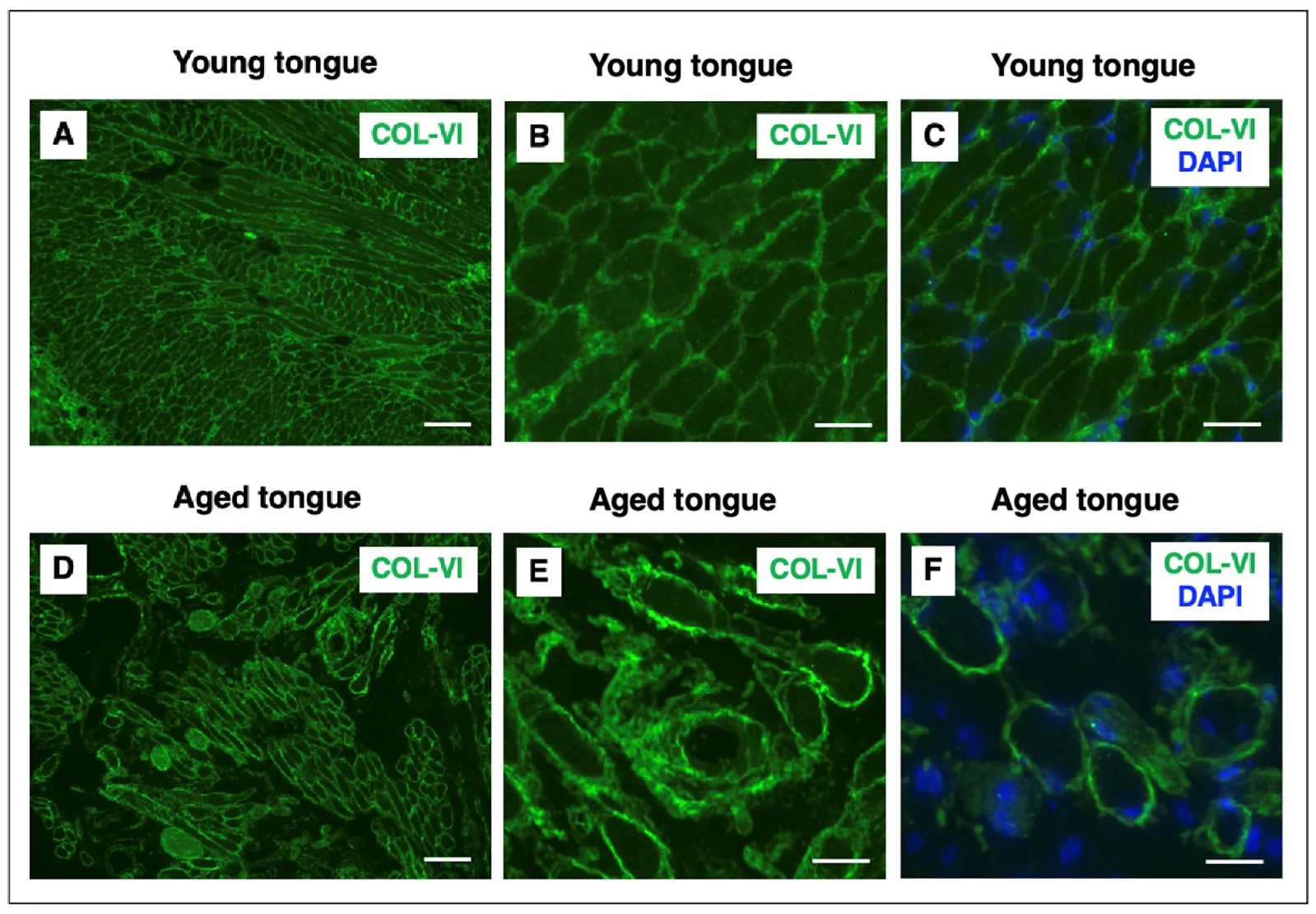
Immunofluorescence microscopical localization of the fibrosis marker collagen isoform COL-VI in young versus aged tongue. Green fluorescence displays labeling of collagen and 4′,6-diamidino-2-phenylindole (DAPI) staining was used to label nuclei. Bars equal 100 μm in figure (**A**,**D**) and 25 μm in figure (**B**,**C**,**E**,**F**).

The above-described proteomic identification of elevated levels of collagen isoform COL-VI suggests increased collagen deposition and a higher degree of susceptibility to reactive myofibrosis in the senescent tongue. This was confirmed by immunofluorescence microscopy of COL-VI as shown in Figure 7. The labeling of the extracellular matrix region clearly shows abnormal cell shapes and diameters of aged myofibers with increased collagen expression (Figure 7D,E) and zones that are partially void of contractile tissues (Figure 7D,F), as compared to young tongue muscle (Figure 7A–C).

## 3. Discussion

This report focuses on the proteomic profiling of the mouse tongue and the determination of age-related changes in the expression profile of its protein constellation. Prior to the comparative proteomic analysis of the senescent tongue, MS-based cataloging of sarcomeric proteins was used to established the broad range of expressed MyHC molecules, including the fast isoforms MyHC-2A (Myosin-2), MyHC-2B (Myosin-4) and MyHC-2X (Myosin-1) [63], as well as embryonic isoform MyHC-3 (Myosin-3) and perinatal isoform MyHC-8 (Myosin-8) [74]. The MyHC-2A (*Myh2* gene) isoform and the MyHC-2X (*Myh1* gene) isoform are established markers of fast oxidative-glycolytic type 2A fibers and fast glycolytic type 2X fibers in matured skeletal muscles, respectively [75]. The additionally identified fast-twitch MyHC-2B (*Myh4* gene) isoform is mostly found in rodent and other non-human muscles [76]. Of note, the superfast isoform MyHC-13 (Myosin-13) was identified in young and aged tongue muscle [77]. The expression of this unusual MyHC is restricted to specialized types of extremely fast-twitching skeletal muscles, including extraocular and laryngeal muscles [64,65]. This study has shown that tongue muscles also belong to this sub-category of voluntary muscles with ultrafast myosin kinetics [63]. Although detailed physiological, transcriptomic, and proteomic surveys of skeletal muscle heterogeneity suggest that multi-dimensional variations exist between myofibers beyond MyHCs [78,79], the general categorization of fast versus slow fiber types based on MyHC distribution patterns is still valid [80]. Hence, the presence of four types of fast MyHC isoforms indicate that a large proportion of fast-twitching myofibers exist in tongue muscles [81]. This agrees with immunohistochemical and molecular biological studies that have shown a high concentration of MyHC-2A and MyHC-2B in predominantly fast-twitching mouse tongue muscles [82]. In contrast to fast MyHCs, slow myosin isoforms appear to be differentially expressed in 3-month versus 24-month old mouse tongue muscles, i.e., cardiac (α) MyHC-6 (Myosin-6) versus slow/cardiac (β) MyHC-1 (Myosin-7), an established marker of slow-oxidative type I myofibers in voluntary striated muscles [63,77].

In addition to myosins, the proteomic survey of tongue muscle identified the main isoforms of the thin filament including actin, nebulin, troponins and tropomyosins, as well as the half-sarcomere spanning protein titin and markers of the M-line and Z-disc [35,36,37,38,39,63]. Organellar markers of ribosomes, the Golgi apparatus, lysosomes, proteasomes, mitochondria, and nuclei were clearly identified [59,60,61], in addition to key components of the sarcosol including glycolytic enzymes [83], metabolite transporters [84] and molecular chaperones [85]. This successful tongue muscle cataloging exercise demonstrated the robustness of the MS-based proteomic approach used in this report, and formed the basis of a comprehensive comparative analysis of the effects of aging on the tongue proteome.

The comparative MS survey of young versus aged tongue preparations revealed interesting findings on the proteome-wide alteration in protein expression levels. The tongue-associated protein with the highest increase was identified as corticosteroid-binding globulin [86], also known as transcortin, which modulates the bioavailability of glucocorticoids and is implicated in the hormonal modulation of muscular atrophy, mitochondrial metabolism, and catabolic protein degradation. Elevated levels of lactadherin, also referred to as milk fat globule epidermal growth factor MFG-E8, which plays a potential anti-inflammatory role, have previously been observed during skeletal muscle aging [87]. Ceruloplasmin is a copper-carrying element that supports iron metabolism and antioxidant defense in skeletal muscles [88]. Increases in inter alpha-trypsin inhibitor agree with previous studies on skeletal muscle aging and its role in inflammation, degeneration, and progressive fibrosis [89]. Additional elevated myofibrosis markers [67] were identified as the FGA, FGB and FFG chains of fibrinogen [90], the small leucine-rich proteoglycan asporin [91] and collagen isoform COL-VI [92]. The excessive deposition of ECM proteins, especially the fibrillar collagen isoform COL-VI, are clear hallmarks of myofibrosis, which were shown to play a central role in loss of tissue elasticity, impaired contractile function and decreased capacity for muscle regeneration, resulting in progressive muscle weakness during aging [93]. Both histological analyses and immunofluorescence microscopical labeling of the general fibrosis marker collagen isoform COL-VI agree with the findings from the systematic proteomic analysis, and suggest fibro-fatty changes within the aging tongue [71,72,73]. This process appears to play a major part in sarcopenia of old age, possibly mediated by fibro-adipogenic progenitors (FAPs) [73] that can differentiate into fibroblasts and adipocytes and trigger intramuscular infiltration of fibrous and adipose tissue. Fibroblast heterogeneity is now an established concept. A variety of cellular phenotypes have been described as contributing to inflammation, fibrosis and adipogenesis in numerous human diseases, as recently reviewed [94], including sarcopenia of old age [73]. The progressive accumulation of non-contractile tissues and tissue scarring related loss of muscle elasticity can drastically impact the normal physiological functioning and adaptability of senescent muscles [19].

Compensatory mechanisms to counteract the aging phenotype might be represented by the increased expression levels of mitochondrial D-beta-hydroxybutyrate dehydrogenase BDH1, adenylate kinase isoform AK4, complement factor CFI, carbonic anhydrase isoform CA1, lysosomal protein palmitoyl-protein thioesterase PPT1, apolipoprotein A-I, carboxypeptidase CPN2, serotransferrin, alpha-2-antiplasmin, the intermediate filament protein desmin, and antithrombin AT-III. Mitochondrial D-beta-hydroxybutyrate dehydrogenase BDH1 is crucial for enhancing mitochondrial bioenergetics by supporting ketone utilization and metabolic flexibility [95]. An increased concentration of adenylate kinase isoform AK4 in aging agrees with its crucial role in the mitochondrial matrix as a stress-responsive protein that regulates cellular bioenergetics via maintaining nucleotide homeostasis [96]. Of note, increased exposure of skeletal muscles to complement factor CFI, an inactivator of the aging-associated complement component C4b, improves muscle regeneration and functional recovery [97]. Carbonic anhydrase CA1 is part of a large arsenal of muscle-associated enzymes that mediates the reversible hydration of carbon dioxide, and is involved in antioxidative protection and the maintenance of intracellular pH in myofibers [98]. The lysosomal protein palmitoyl-protein thioesterase PPT1 is important for muscle differentiation [99]. Apolipoprotein A-I is crucial for efficient glucose uptake in skeletal muscles [100]. Carboxypeptidase CPN2 is a protective subunit of the zinc metalloprotease CPN1 that modulates a variety of protein targets, including M-type creatine kinase (CK-MM) following release from damaged myofibers [101]. Importantly, serotransferrin its receptor are crucial factors in maintaining iron homeostasis in skeletal muscles, which is involved in myofiber metabolism, muscle growth, and regeneration [102]. Alpha-2-antiplasmin, which acts as the primary inhibitor of the enzyme plasmin, was shown to suppresses myoblast fusion, and thus affects myofiber differentiation and muscle repair mechanisms [103]. The increase in desmin, a muscle-specific type III intermediate filament protein [104], confirmed previous studies [105]. Desmin shows an age-related change in phosphorylation levels in skeletal muscles, which might be linked to impaired functioning in relation to force transmission, mechanical stability, and structural integrity in senescent muscles [106]. Antithrombin AT-III can actively suppress muscle damage by reducing inflammatory effects. AT-III was demonstrated to attenuate cellular injury from ischemia-reperfusion in skeletal muscle, making it a protective factor in damaged muscles [107].

In contrast to the above-described elevated levels of tongue proteins, aging was also shown to be associated with a large number of decreased protein species, which might reflect varying degrees of impaired cellular mechanisms, the impact of degenerative processes on the proteome and/or adaptations to adjust to the sarcopenic phenotype in the senescent tongue. Interestingly, the tongue protein with the most decreased abundance was identified as the large ribosomal subunit protein eL22, which agrees with the findings from a previous ribosome profiling analysis of aged human skeletal muscles that identified reduced translation of certain protein species in sarcopenia [108]. In general, a trend of age-related fast-to-slow transitions is observed in individual skeletal muscles [109]. As reported in this study, tongue muscle aging appears to be associated with a certain degree of decreases in fast isoforms of the SERCA1 isoform of the sarcoplasmic reticulum Ca^2+^-ATPase as a marker of calcium handling in predominantly fast-twitching muscles [110] and fast isoform MyHC-2B (Myosin-4) as an indicator of myosin twitch kinetics in fast muscles [24]. This agrees with the pathophysiological concept of preferential degenerative effects on fast-twitching type II fibers during sarcopenia of old age resulting in a slower contracting senescent muscle phenotype [24]. A potential compensatory mechanism is the observed up-regulation of perinatal heavy chain MyHC-8 (Myosin-8) during apparent phases of regeneration in aged myofibers [63].

In analogy to the above discussed increase in collagen isoform COL-VI and associated matrisomal proteins as established markers of myofibrosis [67], there appears to be a major remodeling of the ECM [111] in aged tongue muscles, including decreases in nidogen isoform NID1 of the basal lamina, the matrisomal protein fibulin-5, various laminin subunits, the matricellular protein periostin, thrombospondin TSP-4, and collagen isoforms COL-I and COL-XV. Nidogen NID1 (entactin) [112] is a crucial matrisomal glycoprotein of the basal lamina that is involved in muscle regeneration and cellular adhesion processes [113]. The heterotrimeric glycoprotein laminin-211 complex is highly expressed in skeletal muscles and consists of α2-, β1- and γ1-subunits [114]. The LAMA2/LAMB1/LAMC1 trimer named laminin-211 is centrally located between the sarcolemma and the surrounding matrisomal network mediating cellular proliferation, differentiation, adhesion and myofiber stabilization of contractile fibers [115]. Thus, the age-related reduction in nidogen and the α2- and γ1-subunits of laminin indicate lower levels of key components of the basal lamina that might impair muscle ECM maintenance. The matricellular protein periostin is a crucial component of muscle regeneration [116,117] and the reduced collagen isoforms in aged tongue muscle are the minor form COL-I and the tendon-associated isoform COL-XV [118]. Thrombospondin isoform TSP-4 is an ECM glycoprotein that regulates cell-to-matrix interactions [119].

Besides the ECM, major rearrangements were also indicated to occur in the intracellular cytoskeletal network. Based on the proteomic identification of reduced levels of the intermediate filament type III protein peripherin [120], the intermediate filament type IV protein synemin [121], distinct chains of the microtubules component tubulin [122] and the cytolinker epiplakin [123], tongue aging appears to be associated with considerable cytoskeletal remodeling. Intermediate filament proteins perform crucial bridging functions that anchor the actomyosin apparatus to the surface membrane structure and link costameres to desmin filaments [120,121]. Hence, decreased levels of the cytolinker epiplakin [123] in combination with reduced expression of synemin [121] and peripherin [121] may increase the susceptibility of aged muscles to contraction-induced injury and thereby reduce both the contractile force and mechanical strength of senescent myofibers. Since microtubules are intrinsically involved in the regulation of sarcomeric positioning, muscle mechanics and intracellular transport processes, the altered levels of tubulin chains [122] may reflect degeneration-regeneration cycles in senescent tongue tissues.

The reduction in other types of tongue proteins is potentially connected to abnormal patterns of regeneration, glycosylation, antioxidant response, adaptation of energy metabolism, the innate immune response, iron homeostasis, biotransformation, and blood flow regulation. Latexin is an inhibitor of zinc-dependent metallopeptidases and is involved in muscle satellite cell function, which may affect the rate of regeneration during muscle aging [124]. Mannose-1-phosphate guanylyltransferase catalyzes GDP-mannose formation, which has been shown to be crucial for the proper functioning of the basal lamina component *α*-dystroglycan of the dystrophin complex at costameres which are essential for efficient lateral force transmission [125]. The decrease in tripartite motif-containing protein might have negative effects, since Trim16 was previously shown to assist in the antioxidant response in aged skeletal muscles [126]. Lower levels of the cysteine and glycine-rich protein CSRP3, also known as muscle LIM protein, which is a component of the sarcomeric Z-disc, might affect efficient muscle remodeling of energy metabolism [127]. Protein phosphatase PP1, specifically the listed subunit PP1-R3A, facilitates glycogen metabolism making it a key controller of muscle energy metabolism [128]. Activation of the alternative complement system in the form of complement factor CFD/adipsin is involved in adipocyte-muscle crosstalk, the innate immune response in muscles and adipokine-related effects on skeletal muscle function, which appears to be lowered during aging [129]. Importantly, since serine protease inhibitors function as anti-fibrogenic factors and are crucial for promoting muscle integrity [130], their reduction might impair this response against reactive myofibrosis during aging. Altered levels of ferritin heavy chain FTH1 [131] indicate an age-related disturbance of ion transport in senescent tongue muscle. The considerable change in cytochrome P450 (CYP) levels might influence the efficiency of detoxification of xenobiotics and oxygen homeostasis in aged muscle, since CYP is a crucial enzyme for phase 1 biotransformation reactions, as well as the regulation of blood flow and uptake of oxygen in the neuromuscular system [132]. Several increased proteins, including hexosaminidase B (HEXB), Cathepsin B (CTSB), CD36, coagulation factor XIII A Chain (F13A1) and immunoglobulin chains point toward lysosomal/phagocytic and immune-associated remodeling. However, selected immune-related proteins, including apoptosis-associated speck-like protein containing a CARD (PYCARD/ASC) (inflammasome assembly), complement component 3 (C3) and complement factor D (CFD), show decreased abundance, while complement factor I (CFI) is increased. It looks like aging is associated with a shift in immune-related protein changes rather than a simple global increase in inflammation [133].

Protein biomarkers associated with tongue pressure and biomechanical movements could serve as key clinical functional biomarkers for evaluating swallowing impairment and dysphagia [134]. Tongue pressure biomarkers linked to swallowing difficulties could indicate the early manifestation of neurodegenerative conditions like spinal and bulbar muscular atrophy (SBMA) or amyotrophic lateral sclerosis (ALS). Proteins linked to tongue muscle weakening can become compromised during early or pre-sarcopenic stages as well as indicators of oral frailty in older adults affecting nutritional health and speech production [135].

## 4. Materials and Methods

### 4.1. Materials

For the proteomic profiling of mouse tongue and analysis of its aged phenotype, chemicals and general materials were purchased from Sigma Chemical Company (Dorset, UK), GE Healthcare (Little Chalfont, Buckinghamshire, UK) and Bio-Rad Laboratories (Hemel Hempstead, Hertfordshire, UK). Protease and phosphatase inhibitors, the Pierce 660 nm protein assay and C18 spin columns were obtained from Thermo Fisher Scientific (Hemel Hempstead, Hertfordshire, UK). Mass spectrometry grade trypsin (Trypsin Platinum) and ProteaseMAX surfactant trypsin enhancer were from Promega (Madison, WI, USA). The filter-aided sample preparation method was performed with Vivacon 500 spin filters (VN0H22; 30,000 MWCO) from Sartorius (Göttingen, Germany). The primary antibody against collagen (mAb SD83-03 against collagen VI) was from Invitrogen, Waltham, MA, USA. Fluorescein-conjugated secondary antibodies were purchased from Chemicon International (Temecula, CA, USA).

### 4.2. Ethical Approval, Animal License and Animal Maintenance

Wild type C57/BL6 mice were obtained from the Bioresource Unit of Bonn University and the National University of Ireland, Maynooth. Twelve animals were kept at a constant room temperature of 22 °C on a 12:12 h dark–light cycle with ad libitum access to food and water. All protocols and experiments were performed under the regulations of both the Institutional Animal Care and Use Committee (Germany) and the Department of Agriculture (Ireland). Biochemical and proteomic investigations were exclusively performed with *post mortem* tongue muscle samples. Freshly dissected tongue specimens were quick-frozen in liquid nitrogen and stored at −80 °C prior to proteomic analysis in accordance with the Department of Agriculture (animal by-product register number 2016/16 to the Department of Biology, National University of Ireland, Maynooth) [136].

### 4.3. Preparation of Mouse Tongue Muscle Extracts for Proteomic Analysis

Whole mouse tongue specimens were used in this proteomic study. Muscle protein identification and the comparative analysis of tissue extracts from young (n = 6; 3-month male) versus old (n = 6; 24-month male) wild type mice (C57/BL6) were evaluated in this study [137]. Tissue samples were homogenized in the commonly employed lysis buffer system RIPA (radioimmunoprecipitation assay), which consists of 50 mM Tris-HCl, 150 mM NaCl, 1% NP-40/Triton X-100, 0.5% sodium deoxycholate, 0.1% SDS. A ratio of 1 mL of ice-cold buffer per 50 mg of tissue was used. Following 30 min of incubation on ice and centrifugation at 14,000× *g* for 10 min at 4 °C, the protein-rich supernatant was collected. Importantly, the RIPA buffer contained protease and phosphatase inhibitors (Halt™ Protease and Phosphatase Inhibitor Cocktail, Thermo Scientific, Hemel Hempstead, UK). A Pierce 660 nm protein assay system was used to determine protein concentrations [138]. Buffer exchange of 50 μg of protein lysate was conducted using the filter-aided sample preparation (FASP) method with a buffer containing 8 M urea [139]. After reduction with dithiothreitol and iodoacetic acid-mediated alkylation, digestion was performed using trypsin (Trypsin Platinum, Mass Spectrometry Grade, Promega) overnight at 37 °C using in a buffer containing 50 mM NH_4_HCO_3_/0.1% ProteaseMax. Digested samples were desalted prior to analysis using C18 spin columns (Thermo Scientific, Hemel Hempstead, UK), dried through vacuum centrifugation and resuspended in mass spectrometry loading buffer (2% acetonitrile (ACN), 0.05% trifluoroacetic acid (TFA) in LC-MS grade water). Peptides were vortexed, sonicated and briefly centrifuged at 14,000× *g* and the supernatant transferred to mass spectrometry vials for label-free liquid chromatography mass spectrometry (LFQ LC-MS/MS).

### 4.4. Label-Free Liquid Chromatography Mass Spectrometry

A Thermo Fisher Ascend mass spectrometer coupled with a NeoVanquish LC system was used for label-free liquid chromatography mass spectrometry [137] whereby 500 ng of each digested sample was analyzed. Peptides were eluted over a 90 min binary gradient [solvent A: 2% (*v*/*v*) ACN and 0.1% (*v*/*v*) formic acid in LC-MS grade water and solvent B: 80% (*v*/*v*) ACN and 0.1% (*v*/*v*) formic acid in LC-MS grade water]. Data was acquired with Xcalibur software (Thermo Fisher Scientific). The mass spectrometer was externally calibrated and operated in positive, data-dependent mode. A full survey MS scan was performed in the 300–2000 *m*/*z* range. Collision-induced dissociation (CID) fragmentation was conducted on the fifteen most intense ions per scan (top 15) [140].

### 4.5. Protein Identification and Quantification

Data analysis, processing and visualization for protein identification and label-free quantification (LFQ) normalization of MS/MS data was performed using MaxQuant 2.4.9 (http://www.maxquant.org) and Perseus v2.1.6.0 (www.maxquant.org) software. Significant proteins were initially identified using MaxQuant software and the Andromeda search engine to explore the detected features against the Uni-ProtKB/SwissProt database for Mus musculus (Taxon ID: 10090). The following search parameters were used: (i) first search peptide tolerance of 20 ppm, (ii) main search peptide tolerance of 4.5 ppm, (iii) cysteine carbamidomethylation set as a fixed modification, (iv) methionine oxidation set as a variable modification, (v) a maximum of two missed cleavage sites and (vi) a minimum peptide length of seven amino acids. The false discovery rate (FDR) was set to 1% for both peptides and proteins using a target-decoy approach. Relative quantification was performed using the MaxLFQ algorithm. The “proteinGroups.txt” file produced by MaxQuant was further analyzed in Perseus. Minimum peptide length was set to 7 amino acids to ensure reliable identification. Decoy Mode: A randomized database strategy was used to estimate and filter false discoveries. Enzyme specificity was configured for specific proteolytic cleavage allowing a maximum of 2 missed cleavages. Accurate proteome-wide label-free quantification was processed using delayed normalization and maximal peptide ratio extraction, termed MaxLFQ. A 1.5-fold change in protein abundance was used as the cut-ff threshold for subsequent analysis. Proteins that matched the reverse database or a contaminants database or that were only identified by site were removed. The LFQ intensities were log2 transformed, and proteins with less than 70% valid values were removed from the dataset. Data imputation was performed to replace missing values with values that simulate signals from peptides with low abundance chosen from a normal distribution specified by a downshift of 1.8 times the mean standard deviation of all measured values and a width of 0.3 times this standard deviation. A two-sample *t*-test was performed using *p* < 0.05 on the post-imputated data to identify statistically significant differentially abundant proteins.

All data and analysis files are available on the Open Science Framework (https://osf.io/pq4va/overview?view_only=c3750462773c4513853b1556c5945951; accessed 1 September 2026).

### 4.6. Systems Bioinformatics

Optimized systems bioinformatic tools were employed for the evaluation of proteomic data [141,142], including functional enrichment analyses by ShinyGO with Gene Ontology (GO) terms [68] and Kyoto Encyclopedia of Genes and Genomes (KEGG) [68] pathways. In order to group identified tongue proteins based on their protein class and to identify potential protein–protein interaction patterns, the PANTHER database of protein families [62] (http://pantherdb.org/) and the STRING database of known and putative protein interactions that include both direct physical and indirect functional protein associations [70] (http://string-db.org/) were used [137].

### 4.7. Comparative Histology and Immunofluorescence Microscopy

Histopathological changes in the aged tongue were determined using routine histological hematoxylin and eosin staining and immunofluorescence microscopy, as previously described in detail [143,144]. Freshly dissected tongue muscle specimens from 3-month versus 24-month old mice were quick-frozen in liquid nitrogen-cooled isopentane and 10 µm sections cut in a cryostat. For immuno-labeling of collagen isoform COL-VI, unfixed cryosections were boiled in phosphate-buffered saline for 5 min. Primary antibodies were diluted appropriately in phosphate-buffered saline for overnight incubation at 4 °C. Tongue tissue specimens were washed and incubated with a secondary fluorescently-labeled anti-mouse RRX antibody for 45 min at room temperature. Nuclei were counter-stained with 1 μg/mL 4′,6-diamidino-2-phenylindole (DAPI). Antibody-labeled muscle tissue sections were embedded in Fluoromount G medium and viewed under a Zeiss Axioskop 2 epifluorescence microscope equipped with a digital Zeiss AxioCam HRc camera (Carl Zeiss Jena GmbH, Jena, Germany).

## 5. Conclusions

The MS-based proteomic study of the tongue has established the main protein species of this highly specialized and multifunctional muscular system, which is crucial for taste sensations, mastication, swallowing, clearing of the oral cavity, immune surveillance, speech development, and sensory perception. The expression of established protein markers of the sarcomere and the highly diverse actomyosin apparatus, as well as key organelles and the sarcosol, have been confirmed. Notably, the superfast myosin isoform MyHC-13 was identified in tongue tissue. Lingual muscles therefore belong to the class of very fast-twitching skeletal muscles, such as extraocular and laryngeal muscles, which express this unusual myosin molecule. The comparative analysis of young versus aged tongue has revealed age-related myofibrosis, based on the elevated expression of collagen isoform COL-VI, an established marker of abnormal ECM accumulation in diseased skeletal muscles. In addition, age-related proteomic alterations were identified in proteins that are involved in energy metabolism, hormonal regulation, iron homeostasis, inflammation, mitochondrial flexibility, nucleotide homeostasis, muscle regeneration, carbon dioxide removal, antioxidative protection, glucose metabolism, maintenance of the network of intermediate filaments, cytoskeletal rearrangements, muscle regeneration, protein glycosylation, bioenergetic processes, antioxidant defense, the inflammatory response, iron transport, detoxification and the regulation of blood flow. These findings agree with the pathophysiological concept of considerable changes in the aged tongue proteome and suggest that tongue muscle integrity is impaired during the natural aging process. In analogy to most voluntary striated muscles in the body, sarcopenia of old age seems to also majorly affect the muscle mass and contractile strength of the tongue. The results from the proteomic survey presented in this report confirm organ-wide alterations in the senescent tongue, such as altered tongue pressure, decreased tongue muscle strength, elevated fat mass, stem cell exhaustion, bioenergetic alterations, fiber type shifting and reactive myofibrosis. Hence, the application of rigorous quantitative LC-MS/MS resulted in the biochemical establishment of a comprehensive list of protein alterations in the aged tongue. The future orthogonal validation of these novel proteomic findings using biochemical assays, physiological measurement and immunochemical analyses can provide verification of protein abundance changes and new mechanistic insights into the aging process. However, these types of analyses are beyond the scope of this initial MS-based investigation, which was designed as a discovery-based proteomic analysis.

## Figures and Tables

**Table 1:** List of major sarcomeric tongue muscle proteins of the actomyosin apparatus, the titin filament, the Z-disc, and the M-line as determined by MS-based proteomics.

Name of Tongue Muscle Protein (Molecular Mass; Gene Name)	Protein Accession Number	Number of Peptides	Sequence Coverage (%)	Number of Peptides	Sequence Coverage (%)
		Young Tongue		Aged Tongue	
**Contractile proteins of the myosin-containing thick filament**					
Myosin-1; fast myosin heavy chain MyHC-2X, fast (223.3 kDa; *Myh1*)	Q5SX40	138	60.3	135	60.2
Myosin-2; fast myosin heavy chain MyHC-2A, fast (223.2 kDa; *Myh2*)	G3UW82|	105	43.3	103	43.1
Myosin-3, embryonic myosin heavy chain MyHC-3 (223.6 kDa; *Myh3*)	P13541	51	21.2	47	19.9
Myosin-4; fast myosin heavy chain MyHC-2B (222.9 kDa; *Myh4*)	Q5SX39	148	64.6	144	64.3
Myosin-6; cardiac (a) myosin heavy chain MyHC-6 (223.6 kDa; *Myh6*)	Q02566	43	17.0	-	-
Myosin-7; slow/cardiac (b) myosin heavy chain MyHC-1 (222.9 kDa; *Myh7*)	Q91Z83	-	-	38	16.7
Myosin-8; perinatal myosin heavy chain MyHC-8 (222.7 kDa; *Myh8*)	P13542	89	37.3	87	36.8
Myosin-13; fast myosin heavy chain MyHC-13 (223.6 kDa; *Myh13*)	B1AR69	44	18.8	38	17.4
Myosin light chain MLC1/3, essential MLC, fast (17.7 kDa; *Myl1*)	E9PWG4	13	69.6	13	69.6
Myosin light chain MLC3, essential MLC, slow (22.4 kDa; *Myl3*)	P09542	7	36.3	-	-
Myosin light chain MLC11, regulatory MLC, fast (18.9 kDa; *Mylpf/Myl11*)	P97457	17	92.9	17	92.9
Myosin light chain kinase MYLK2 (65.9 kDa; *Mylk2*)	Q8VCR8	8	21.2	9	24.8
Myosin binding protein MyBP-C, slow (126.6 kDa; *Mybpc1*)	D3YU50	63	66.8	70	68.4
Myosin binding protein MyBP-C, fast (127.4 kDa; *Mybpc2*)	Q5XKE0	17	22.1	11	12.1
**Contractile and regulatory proteins of the actin-containing thin filament**					
Actin, alpha, muscle (42.1 kDa; *Acta1*)	P68134	21	70.0	23	70.8
Troponin TnC (18.1 kDa; *Tnnc2*)	P20801	11	73.1	9	62.5
Troponin TnI (21.4 kDa; *Tnni2*)	P13412	13	51.6	14	56.6
Troponin TnT (32.2 kDa; *Tnnt3*)	Q9QZ47	13	37.5	12	36.8
Tropomyosin TPM1 (28.3 kDa; *Tpm1*)	E9Q456	16	49.8	16	49.6
Tropomyosin TPM2 (32.8 kDa; *Tpm2*)	P58774	27	70.1	27	70.1
Tropomyosin TPM3 (28.7 kDa; *Tpm3*)	Q9WTX6	19	52.2	24	64.5
Tropomyosin TPM4 (28.5 kDa; *Tpm4*)	Q6IRU2	21	66.1	21	68.1
F-actin-capping protein CapZ-A1, alpha-1 (32.9 kDa; *Capza1*)	P47753	6	34.6	5	27.6
F-actin-capping protein CapZ-A2, alpha-2 (32.9 kDa; *Capza2*)	P47754|	12	64.0	12	64.0
F-actin-capping protein CapZ-B, beta (31.4 kDa; *Capzb*)	A2AMW0	12	49.2	11	43.7
Tropomodulin TMOD1 (40.5 kDa; *Tmod1*)	P49813	5	16.7	6	21.2
Tropomodulin TMOD3 (39.5 kDa; *Tmod3*)	Q9JHJ0	3	10.8	3	7.7
Xin actin-binding protein XIRP (196.7 kDa; *Xirp1*)	O70373	18	18.2	12	9.2
Myopalladin MYPN (144.1 kDa; *Mypn*)	Q5DTJ9	7	7.1	7	8.2
Nebulin NEB (871.2 kDa, *Neb*)	A0A571BF58	282	50.5	264	48.4
Nebulin-related anchoring protein NRAP (195.8 kDa; *Nrap*)	Q80XB4	23	16.7	15	10.6
**Proteins of the auxiliary titin filament**					
Titin (3925.5 kDa; *Ttn*)	A0A5K1VVQ9	1171	44.7	1167	44.3
Titin-associated muscle ankyrin repeat protein (37.1 kDa; *Ankrd2*)	A0A494B9J0	3	9.6	5	20.8
**Sarcomeric proteins of the Z-disc**					
Actinin-2, alpha (103.8 kDa; *Actn2*)	Q9JI91	54	75.4	55	75.5
Myozenin (31.5 kDa; *Myoz1*)	Q9JK37	10	54.1	11	56.4
Filamin-C, muscle (291.1 kDa; *Flnc*)	Q8VHX6	78	40.5	72	37.0
Telethonin TCAP (19.1 kDa; *Tcap*)	A0A0A0MQN1	3	19.2	2	12.6
**Sarcomeric proteins of the M-line**					
Obscurin (966.6 kDa; *Obscn*)	A2AAJ9	173	37.2	170	35.9
Myomesin-1 (185.5 Da; *Myom1*)	Q62234	84	63.6	84	62.6
Myomesin-2 (164.8 kDa; *Myom2*)	Q14BI5	77	64.2	78	63.0
Myomesin-3 (161.8 kDa; *Myom3*)	A2ABU4	44	45.3	48	46.8

**Table 2:** List of subcellular marker proteins as determined by MS-based proteomics.

Tongue Protein Name (Protein Accession Number; Molecular Mass; Gene Name)	Subcellular Localization	Number of Peptides	Sequence Coverage (%)	Number of Peptides	Sequence Coverage (%)
		Young Tongue		Aged Tongue	
Albumin (P07724; 68.7 kDa; *Alb*)	Extracellular space	58	83.1	64	83.6
Hemoglobin, α-globin (Q91VB8; 15.1 kDa; *Hba*)	External oxygen supply	10	90.1	9	90.1
Collagen COL-I (P11087; 138.0 kDa; *Col1a1*)	Epimysium	8	6.0	4	3.2
Collagen COL-VI, myofibrillar (J3QQ16; 288.7 kDa; *Col6a3*)	Endomysium	63	31.8	68	33.0
Decorin (P28654; 39.8 kDa; *Dcn*)	Perimysium	13	43.5	14	47.2
Laminin, α2-subunit (Q60675; 233.4 kDa; *Lama2*)	Basal lamina	46	23.0	24	11.4
Integrin, α7-subunit (G3X9Q1; 124.0 kDa; *Itga7*)	Sarcolemmal adhesion	16	19.3	15	18.3
Talin (P26039; 269.8 kDa; *Tln1*)	Costameres	79	45.1	72	41.0
Na^+^/K^+^-ATPase, α1-subunit (Q8VDN2; 112.9 kDa; *Atp1a1*)	Sarcolemma	37	43.2	38	44.0
Caveolin CAV1 (P49817; 51.7 kDa; *Cav1*)	Caveolae	11	68.0	9	51.1
Dysferlin (E9QL12; 237.2 kDa; *Dysf*)	Sarcolemmal repair system	32	26.3	34	24.4
Voltage-dependent L-type Ca^2+^- channel, α1S-subunit (A0A8I4RSM0; 210.2 kDa; *Cacna1s*)	Transverse tubules	21	13.1	17	9.9
Spectrin, βIΣ2-isoform (Q3UGX2; 268.1 kDa; *Sptb*)	Membrane cytoskeleton	57	42.5	63	35.8
Desmin (P31001; 53.5 kDa; *Des*)	Intermediate filaments	30	63.1	33	70.6
Tubulin, b-4B (P68372; 49.8 kDa; *Tubb4b*)	Microtubules	23	71.0	24	71.0
Ca^2+^-ATPase, fast, SERCA1 (Q8R429; 109.4 kDa; *Atp2a1*)	Sarcoplasmic reticulum, longitudinal	58	54.9	59	55.4
Calsequestrin, fast, CSQ1-isoform (O09165; 46.4 kDa; *Casq1*)	Sarcoplasmic reticulum, terminal cisternae	20	64.4	19	64.4
Sarcalumenin (Q7TQ48; 54.3. kDa; *Srl*)	Sarcoplasmic reticulum, lumen	31	69.9	34	72.7
Ryanodine receptor Ca^2+^-release channel, RyR1-isoform (E9PZQ0; 565.0 kDa; *Ryr1*)	Triad junction membrane	156	44.9	168	47.5
40S ribosomal protein S4 (P62702; 29.6 kDa; *Rps4x*)	Ribosome	13	44.9	14	49.4
Vesicular transport factor (Q9Z1Z0; 106.9 kDa; *Uso1*)	Golgi apparatus	21	32.3	21	31.4
Lysosome-associated membrane glycoprotein LAMP1 (P11438; 43.9 kDa; *Lamp1*)	Lysosome	3	8.4	3	8.4
Catalase (P24270; 59.8 kDa; *Cat*)	Peroxisome	19	49.1	19	47.1
Ubiquitin-conjugating enzyme E2 (P61089; 17.1 kDa; Ube2n)	Proteasome	10	80.9	10	80.9
Emerin (O08579; 29.4 kDa; *Emd*)	Nucleus	6	29.7	2	17.1
Voltage-dependent anion-selective channel protein VDAC1 (Q60932; 28.2 kDa; *Vdac1*)	Mitochondrium, outer membrane	21	79.1	20	78.7
MIC60 subunit of mitochondrial contact site complex (E9Q800; 75.6 kDa; *Immt*)	Mitochondrial contact sites, MICOS complex	46	67.9	50	71.1
NADH dehydrogenase (Q8K3J1; 24.0 kDa; *Ndufs8*)	Mitochondrium, inner membrane complex I	9	57.5	10	62.7
Succinate dehydrogenase (Q8K2B3; 72.6 kDa; *Sdha*)	Mitochondrium, inner membrane complex II	38	78.3	37	74.1
Cytochrome b-c complex (Q9CQB4; 13.6 kDa; *Uqcrb*)	Mitochondrium, inner membrane complex III	11	63.1	11	59.5
Cytochrome c oxidase (P00405; 25.9 kDa; *Mtco2*)	Mitochondrium, inner membrane complex IV	9	60.8	8	60.8
ATP synthase, *a*-subunit (Q03265; 59.8 kDa; *Atp5f1a*)	Mitochondrium, inner membrane complex V	38	67.8	37	67.6
Isocitrate dehydrogenase IDH2 (P54071; 50.9 kDa; *Idh2*)	Mitochondrium, matrix	28	58.8	30	68.4
Hexokinase isoform HK2 (O08528; 102.5 kDa; *Hk2*)	Metabolic flux regulator between mitochondrium and cytosol	43	52.9	43	54.5
Phosphofructokinase, muscle-type (P47857; 85.3 kDa; *Pfkm*)	Cytosol, glycolytic pathway	43	74.5	42	73.8
Fatty acid binding protein, FABP3/FABPH (P11404; 14.8 kDa; *Fabp3*)	Cytosol, metabolite transportation	12	79.7	12	84.2
Myoglobin (P04247; 17.1 kDa; *Mb*)	Intracellular oxygen transport	22	86.4	22	91.6
Cardiovascular heat shock protein HSPB7 (P35385; 18.6 kDa; *Hsbp7*)	Cytosol, cellular stress response	9	68.6	8	79.9

**Table 3:** List of identified proteins with an increased abundance in aged mouse tongue that are relevant to sarcopenia of old age.

Tongue Protein Name (Molecular Mass)	Accession Number	Gene Name	Peptides	Coverage (%)	ANOVA	Fold Change
Corticosteroid-binding globulin, transcortin (44.8 kDa)	Q06770	*Serpina6*	13	37.3	0.00185	6.2
Lactadherin MFG-E8 (51.2 kDa)	P21956	*Mfge8*	12	32	0.00782	3.0
Ceruloplasmin (121.1 kDa)	G3X9T8	*Cp*	54	58.2	0.00101	2.9
Inter alpha-trypsin inhibitor, heavy chain 4 (104.7 kDa)	A6X935	*Itih4*	18	27.5	0.00319	2.1
D-beta-hydroxybutyrate dehydrogenase BDH1, mitochondrial (38.3 kDa)	Q80XN0	*Bdh1*	14	51.9	2.12 × 10^−5^	2.0
Fibrinogen, gamma chain FGG (49.4 kDa)	Q8VCM7	*Fgg*	17	52.3	0.00423	2.0
Fibrinogen, beta chain FGB (54.8 kDa)	Q8K0E8	*Fgb*	19	54.9	0.00404	2.0
Complement factor CFI (67.3 kDa)	Q61129	*Cfi*	16	29.4	6.29 × 10^−5^	1.9
Fibrinogen, alpha chain FGA (87.4 kDa)	E9PV24	*Fga*	20	32.7	0.00060	1.9
Adenylate kinase AK4, mitochondrial (25.1 kDa)	Q9WUR9	*Ak4*	10	58.3	0.00033	1.8
Carbonic anhydrase CA1 (28.3 kDa)	P13634	*Ca1*	12	75.9	0.01468	1.7
Palmitoyl-protein thioesterase PPT1 (34.5 kDa)	O88531	*Ppt1*	6	28.4	0.01895	1.6
Apolipoprotein A-I (30.6 kDa)	Q00623	*Apoa1*	15	58.3	0.00896	1.5
Carboxypeptidase CPN2 (60.5 kDa)	Q9DBB9	*Cpn2*	8	21.8	0.04121	1.5
Collagen COL-VI, alpha-2 (VI) chain (110.6 kDa)	Q02788	*Col6a2*	20	26.0	0.00429	1.5
Serotransferrin (76.7 kDa)	Q921I1	*Tf*	54	66.9	0.00025	1.5
Alpha-2-antiplasmin (54.9 kDa)	Q61247	*Serpinf2*	9	27.3	0.01059	1.5
Desmin (53.5 kDa)	P31001	*Des*	31	67.8	0.00928	1.5
Antithrombin AT-III (52.0 kDa)	P32261	*Serpinc1*	21	49.0	0.00102	1.5
Asporin (42.6 kDa)	Q99MQ4	*Aspn*	15	47.2	0.00018	1.5

**Table 4:** List of identified proteins with a decreased abundance in aged mouse tongue that are relevant to sarcopenia of old age.

Tongue Protein Name (Molecular Mass)	Accession Number	Gene Name	Peptides	Coverage (%)	ANOVA	Fold Change
Ca^2+^-ATPase, fast, SERCA1 (109.4 kDa)	F6RQN3	*Atp2a1*	4	24.7	0.007396	1.5
Latexin (25.5 kDa)	P70202	*Lxn*	2	13.5	0.02967	1.5
Mannose-1-phosphate guanylyltransferase, a-subunit (46.2 kDa)	Q922H4	*Gmppa*	5	14.3	0.003213	1.5
Nidogen NID1 (136.5 kDa)	P10493	*Nid1*	36	41	0.004242	1.5
Myosin-4; fast heavy chain MyHC-2B (222.9 kDa)	Q5SX39	*Myh4*	146	64	0.047352	1.5
Cysteine and glycine-rich protein CSRP3 (20.8 kDa)	P50462	*Csrp3*	10	58.8	0.043775	1.5
Synemin (173.2 kDa)	Q70IV5	*Synm*	12	12.2	0.01824	1.5
Tripartite motif-containing protein Trim16 (62.9 kDa)	Q99PP9	*Trim16*	6	13.3	0.045298	1.6
Protein phosphatase PP1, subunit 3A (121.4 kDa)	Q99MR9	*Ppp1r3a*	26	34.4	0.000766	1.6
Periostin (93.1 kDa)	Q62009	*Postn*	15	24	0.009231	1.6
Complement factor CFD (28.1 kDa)	P03953	*Cfd*	6	41.7	0.000158	1.6
Laminin, subunit gamma-1 (177.2 kDa)	F8VQJ3	*Lamc1*	39	38.6	0.002726	1.7
Protein FAM25A (9.2 kDa)	Q8CF02	*Fam25c*	3	53.9	0.006929	1.7
Peripherin (54.3 kDa)	G5E846	*Prph*	13	29.5	0.037913	1.7
Myosin-8; perinatal heavy chain MyHC-8 (222.7 kDa)	P13542	*Myh8*	91	37.5	0.015239	1.7
Laminin, subunit alpha-4 (201.8 kDa)	P97927	*Lama4*	6	4.7	0.016974	1.7
Tubulin, beta-3 chain (50.4 kDa)	Q9ERD7	*Tubb3*	18	49.1	0.020752	1.8
Collagen COL-I, alpha-1 (I) chain (138.0 kDa)	P11087	*Col1a1*	7	5.4	0.010113	1.8
Fibulin-5 (50.2 kDa)	Q9WVH9	*Fbln5*	11	33	6.18 × 10^−5^	1.8
Collagen COL-XV, alpha-1 (XV) chain (138.3 kDa)	A2AJY2	*Col15a1*	10	9.1	0.003328	1.8
Serine protease inhibitor A3K (46.7 kDa)	A0A0R4J0I1	*Serpina3k*	20	51.2	0.032397	1.8
Thrombospondin TSP4 (106.4 kDa)	Q9Z1T2	*Thbs4*	14	23.9	0.000116	1.9
Demilune cell and parotid protein DCPP1 (18.6 kDa)	L7N1X9	*Dcpp1*	6	53.2	0.002022	1.9
Laminin, subunit alpha-2 (343.8 kDa)	Q60675	*Lama2*	46	23.2	0.007731	2
Malectin (32.3 kDa)	Q6ZQI3	*Mlec*	6	27.5	0.016647	2.1
Epiplakin (723.3 kDa)	Q8R0W0	*Eppk1*	35	36.1	0.005323	2.2
Tubulin, alpha-8 chain (50.1 kDa)	Q9JJZ2	*Tuba8*	17	47.9	0.025497	3
Murinoglobulin MUG1 (165.3 kDa)	P28665	*Mug1*	46	42.3	0.032629	4.1
Ferritin heavy chain FTH1 (21.1 kDa)	P09528	*Fth1*	5	30.8	0.030354	5.7
Cytochrome P450, CYP (61.9 kDa)	Q8BGU0	*Cyp4f39*	6	14.7	0.02190	7.3
Large ribosomal subunit protein eL22 (14.8 kDa)	P67984	*Rpl22*	3	39.1	0.02530	17.9

## Data Availability

The original data presented in the study is available in the Open Science Framework (OSF) at http://osf.io/pq4va/overview?view_only=c3750462773c4513853b1556c5945951; accessed on 1 September 2026.

## Supplementary Materials

The following supporting information can be downloaded at: https://www.mdpi.com/article/10.3390/muscles5040073/s1, Table S1: Protein list increased in abundance in 3-month vs. 24-month; Table S2: Protein list increased in abundance in 24-month vs. 3-month.

## Abbreviations

The following abbreviations are used in this manuscript:

ACTA	α-Actin, muscle
ACTN	α-Actinin
AK	Adenylate kinase
ANKRD	Ankyrin repeat protein
AT	Antithrombin
BDH	D-beta-hydroxybutyrate dehydrogenase
CA	Carbonic anhydrase
CapZ	F-actin capping protein
CAV	Caveolin
CF	Complement factor
COL	Collagen
CPN	Carboxypeptidase
CSQ	Calsequestrin
CSRP	Cysteine and glycine-rich protein
CY	Cytochrome
DAPI	4′,6-Diamidino-2-phenylindole
DCPP	Demilune cell and parotid protein
ECM	Extracellular matrix
FABP	Fatty acid binding protein
FGA	Fibrinogen, alpha
FGB	Fibrinogen, beta
FGG	Fibrinogen, gamma
FLNC	Filamin-C, muscle
FTH	Ferritin heavy chain
HK	Hexokinase
HSP	Heat shock protein
IDH	Isocitrate dehydrogenase
LAM	Laminin
LAMP	Lysosome-associated membrane glycoprotein
LC	Liquid chromatography
LC-MS/MS	Liquid chromatography tandem mass spectrometry
LFQ	Label-free quantification
MLC	Myosin light chain
MS	Mass spectrometry
MUG	Murinoglobulin
MyBP	Myosin binding protein
MyHC	Myosin heavy chain
MYLK	Myosin light chain kinase
MYOM	Myomezin
MYOZ	Myozenin
MYPN	Myopalladin
NEB	Nebulin
NID	Nidogen
NRAP	Nebulin-related anchoring protein
OBSCN	Obscurin
OSF	Open Science Framework
PP	Protein phosphatase
PPT	Palmitoyl-protein thioesterase
RyR	Ryanodine receptor
TCAP	Telethonin
TSP	Thrombospondin
TMOD	Tropmodulin
TnC	Troponin, calcium-binding subunit
TnI	Troponin, inhibitory subunit
TnT	Troponin, tropomyosin-binding subunit
TPM	Tropomyosin
TRIM	Tripartite motif-containing protein
TTN	Titin
SERCA	Sarcoplasmic or endoplasmic reticulum calcium ATPase
XIRP	Xin actin-binding protein
